# Manganese carbonate nanoparticles‐mediated mitochondrial dysfunction for enhanced sonodynamic therapy

**DOI:** 10.1002/EXP.20210010

**Published:** 2021-09-30

**Authors:** Haoyuan Zhang, Xueting Pan, Qingyuan Wu, Juan Guo, Chaohui Wang, Huiyu Liu

**Affiliations:** ^1^ Beijing Advanced Innovation Center for Soft Matter Science and Engineering, State Key Laboratory of Organic–Inorganic Composites, Beijing Laboratory of Biomedical Materials, Bionanomaterials & Translational Engineering Laboratory, Beijing Key Laboratory of Bioprocess Beijing University of Chemical Technology Beijing P. R. China

**Keywords:** manganese carbonate, mitochondrial regulation, sonodynamic therapy, sonosensitizer, ultrasonic cavitation

## Abstract

Sonodynamic therapy (SDT) has attracted widespread attention due to its non‐invasiveness and deep tissue penetration. However, the development of efficient sonodynamic nanoplatforms to improve the therapeutic efficiency is still one of the main challenges of current research. In this work, a new type of sonosensitizer prepared by a simple method, manganese carbonate nanoparticles (MnCO_3_ NPs), is used for enhanced SDT. MnCO_3_ NPs could generate large amounts of ^1^O_2_ and •OH under ultrasound irradiation. At the same time, CO_2_ and Mn ions could be released in a weak acid environment due to the excellent degradability of MnCO_3_ NPs. The CO_2_ bubbles caused cell necrosis by ultrasonic cavitation and used for ultrasound imaging. And Mn ions activated the mitochondrial cell apoptosis pathway. In vivo experiments proved that this sonosensitizer with mitochondrial regulatory capacity showed high tumor inhibition rates for enhanced sonodynamic tumor therapy.

## Graphical abstract

The cubic manganese carbonate nanoparticles (MnCO_3_ NPs) are prepared by a simple synthesis method for enhanced sonodynamic therapy. It is proved that the high sonodynamic performance of MnCO_3_ NPs is related to its band gap. The CO_2_ and Mn ions produced by the degradation can achieve ultrasonic cavitation and activate the mitochondrial cell apoptosis pathway, respectively. This work provides a new avenue for the development of nanotheranostics.

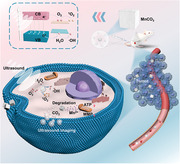

## 1 INTRODUCTION

In recent years, light‐induced photothermal therapy and photodynamic therapy have made major scientific breakthroughs in tumor treatment.^[^ ^1^ ^]^ However, phototherapy is not adequate for deep tumors due to the limited penetration depth of the light.^[^ ^2^ ^]^ Sonodynamic therapy (SDT) is an emerging treatment strategy that uses ultrasound (US) as the excitation source. It has attracted widespread interest because of its non‐invasiveness and deep tissue penetration.^[^ ^3^ ^]^ Currently, one of its research hotspots is the development and utilization of high‐performance sonosensitizers.^[^ ^4^ ^]^ Sonosensitizers can generally be divided into two categories: organic and inorganic sonosensitizers.^[^ ^5^ ^]^ Organic sonosensitizers are mainly hydrophobic small molecules, such as porphyrin^[^ ^6^ ^]^ and its derivatives,^[^ ^7^ ^]^ cyanine dye,^[^ ^8^ ^]^ chlorophyll derivative.^[^ ^9^ ^]^ Due to its poor stability and low bioavailability, resulting in unsatisfactory therapeutic effect.^[^ ^10^ ^]^ Compared with organic sonosensitizers, TiO_2_, a representative inorganic sonosensitizers,^[^ ^11^ ^]^ has better chemical stability and water solubility. However, its further application in SDT is hindered by its difficult metabolism and fast electron‐hole recombination rate.^[^ ^12^ ^]^

The common ways to improve the efficiency of SDT are as follows: (1) relieve tumor hypoxic microenvironment;^[^ ^13^ ^]^ (2) enhance the cavitation effect of sonosensitizers;^[^ ^14^ ^]^ (3) combine SDT with therapeutic methods (chemotherapy,^[^ ^15^ ^]^ photothermal therapy,^[^ ^16^ ^]^ chemodynamic therapy,^[^ ^17^ ^]^ immunotherapy,^[^ ^18^ ^]^ etc.). Recent studies have shown that sonosensitizer mediated‐ultrasonic cavitation not only promotes the generation of reactive oxygen species (ROS), but also induces mechanical force, which directly kills cancer cells.^[^ ^19^ ^]^ Therefore, the enhancement of the cavitation effect of sonosensitizers is expected to achieve better therapeutic effects. In addition, mitochondria are the main place where adenosine triphosphate (ATP) is produced in cells. The dysfunction of mitochondria will affect the catabolic processes of cells, including apoptosis, necrosis, and autophagy. The combination of sonodynamic and mitochondrial regulation is a promising approach to eradicate cancer cells.^[^ ^20^ ^]^ In the past few decades, a variety of strategies have been proposed for cancer treatment by activating the mitochondrial‐induced apoptotic pathway.^[^ ^21^ ^]^ Among them, ion therapy is one of the most effective methods. Ca^2+[^ ^22^ ^]^ and Ag^+^^[^ ^23^ ^]^ can cause mitochondrial dysfunction, reducing mitochondrial membrane potential and intracellular ATP levels. In addition, it is reported that Mn^2+^ has the ability to regulate mitochondrial function and induce apoptosis,^[^ ^24^ ^]^ but there are few studies on Mn^2+^ mitochondrial ion therapy. The above‐mentioned treatment strategies can effectively improve the treatment outcome, but the preparation of nanoparticles often requires more complicated design and assembly. And in the current situation, the mechanism of SDT is still controversial, the complex system is not friendly to the research on the principle of SDT.

**SCHEME 1:**
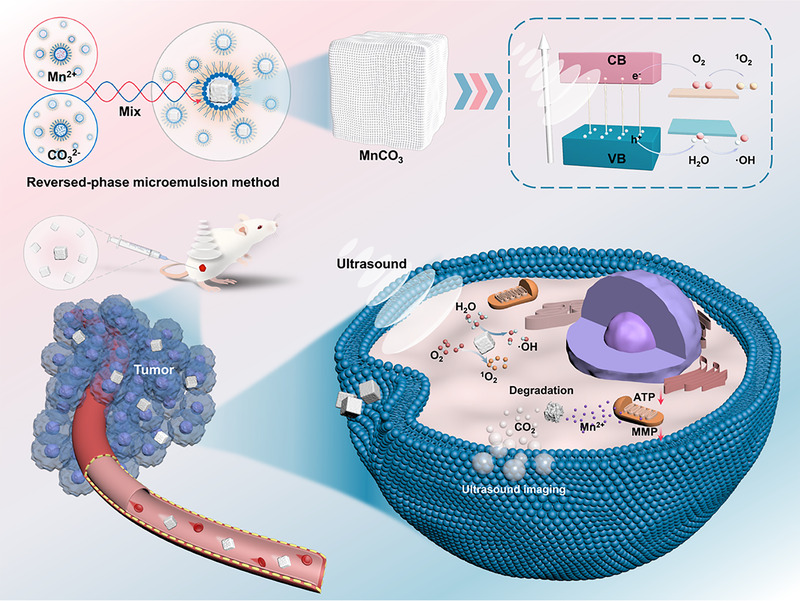
Schematic diagram of the synthesis and antitumor therapy of MnCO_3_ NPs

Herein, we developed a novel sonosensitizer, manganese carbonate nanoparticles (MnCO_3_ NPs), for enhanced SDT (Scheme 1). The MnCO_3_ NPs were synthesized by the inverse microemulsion method. Under US irradiation, MnCO_3_ NPs can efficiently produce hydroxyl radicals (•OH) and singlet oxygen (^1^O_2_). Moreover, MnCO_3_ NPs will release CO_2_ and Mn^2+^ due to the degradation caused by the local acidic microenvironment. The generated CO_2_ bubbles will be triggered explosion by US waves, resulting in irreversible cell necrosis. Meanwhile, the release of Mn^2+^ could induce cell apoptosis by causing mitochondrial dysfunction. Furthermore, the MnCO_3_ NPs exhibit excellent US imaging contrast capability for SDT guidance because of the release of CO_2_. In vivo experiments proved that MnCO_3_ NPs have a tumor inhibition rate of 50.41%, and a higher inhibition (90.45%) on tumor progression is achieved after US irradiation. We believe that this SDT synergistic anti‐cancer strategy could provide new ideas and insights for the development of nanotheranostics.

## 2 RESULTS AND DISCUSSION

### 2.1 Preparation and characterization of MnCO_3_ NPs

**FIGURE 1:**
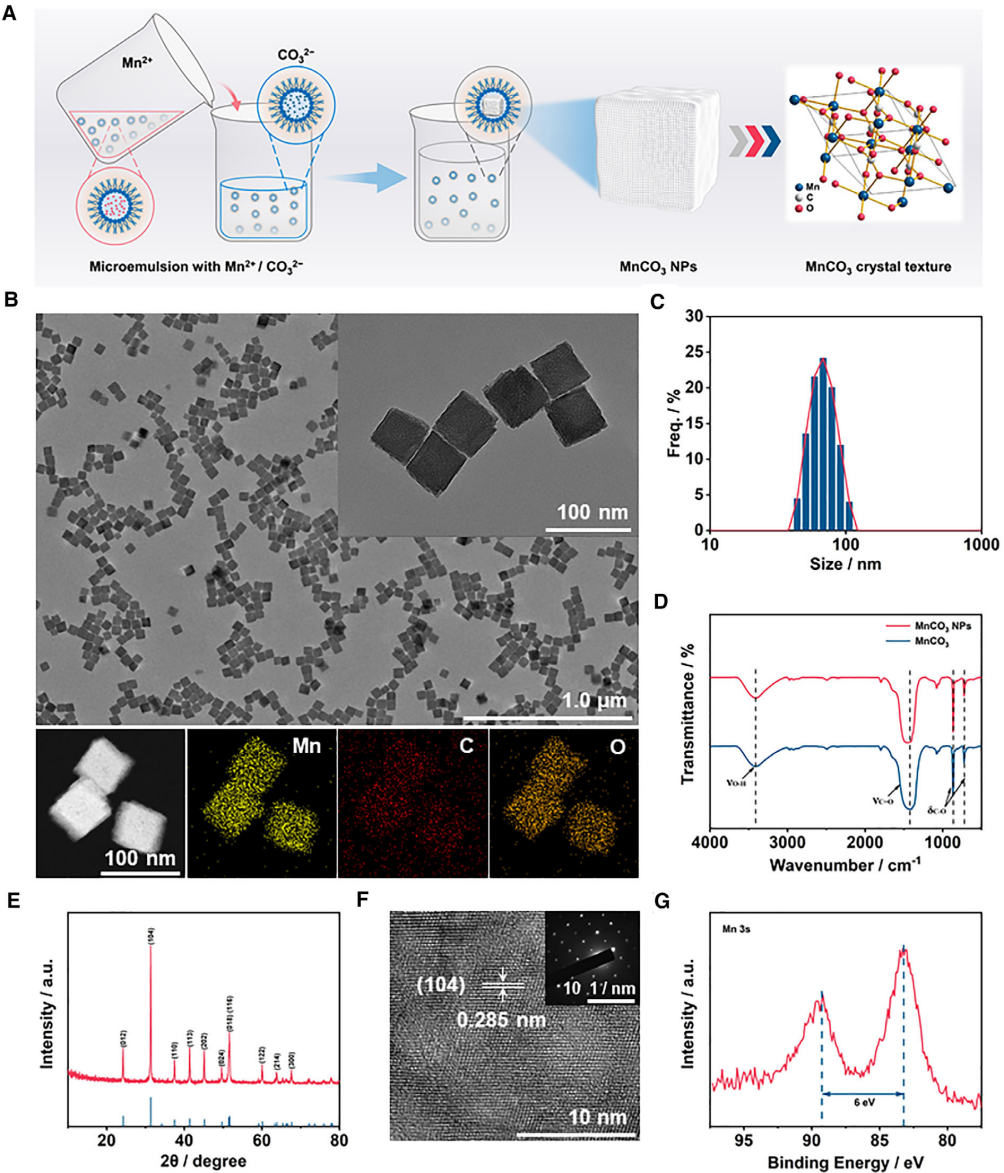
Characterization of MnCO_3_ NPs. (A) Synthesis scheme of MnCO_3_ NPs. (B) TEM images of MnCO_3_ NPs and element mapping for manganese, carbon, oxygen of MnCO_3_ NPs. (C) The size distribution of MnCO_3_ NPs. (D) FT‐IR spectra of MnCO_3_ NPs and MnCO_3_. (E) XRD pattern of MnCO_3_ NPs. (F) HAADF‐STEM image of MnCO_3_ NPs. (G) XPS spectrum of Mn 3s peaks in MnCO_3_ NPs

The synthetic process for MnCO_3_ NPs is illustrated in Figure 1A. MnCO_3_ NPs were synthesized by the inverse microemulsion method according to previous report with slight modifications.^[^ ^25^ ^]^ Transmission electron microscopy (TEM), scanning electron microscope (SEM), and dynamic light scattering (DLS) were used to characterize the morphology and size of prepared MnCO_3_ NPs. The uniform and dispersed MnCO_3_ NPs can be clearly observed under TEM image (Figure 1B). Observation by SEM image confirmed that the MnCO_3_ NPs were cubic structure (Figure S1). And the corresponding energy‐dispersive X‐ray spectroscopy (EDS) element mapping showed the existence of Mn, C, and O elements in MnCO_3_ NPs (Figure 1B and Figure S2). The MnCO_3_ NPs can be well dispersed in water with an average hydrated diameter of 75 nm determined by DLS (Figure 1C). The zeta potential is +19.8 mV (Figure S3). Figure 1D represents the Fourier transform infrared (FT‐IR) spectra of MnCO_3_ NPs. The peaks centered at 725, 860, and 1442 cm^−1^ are the characteristic peaks of MnCO_3_.^[^ ^26^ ^]^ The crystalline structures were characterized by X‐ray diffraction (XRD), all characteristic peaks were consistent with standard powder MnCO_3_ (JCPDS No. 44–1472) (Figure 1E). High‐angle annular dark‐field scanning transmission electron microscopy (HAADF‐STEM) image is shown in Figure 1F. It is clearly revealed that the lattice fringes of MnCO_3_, and the detected lattice fringe of 0.285 nm corresponds to the (104) diffraction plane of MnCO_3_. By analyzing the X‐ray photoelectron spectroscopy (XPS) spectrum, it can be determined that the MnCO_3_ NPs are mainly Mn^2+^ (Figure 1G and Figure S4). The content of Mn in MnCO_3_ NPs was measured to be about 47.75% (Figure S5) by inductively coupled plasma mass spectrometry (ICP‐MS). Thermogravimetric analysis indicated that MnCO_3_ NPs had good stability within 300°C (Figure S6). In conclusion, the above results indicated the successful preparation of structurally well‐defined MnCO_3_ NPs with uniform morphology.

### 2.2 Sonodynamic performance and mechanism of MnCO_3_ NPs

**FIGURE 2:**
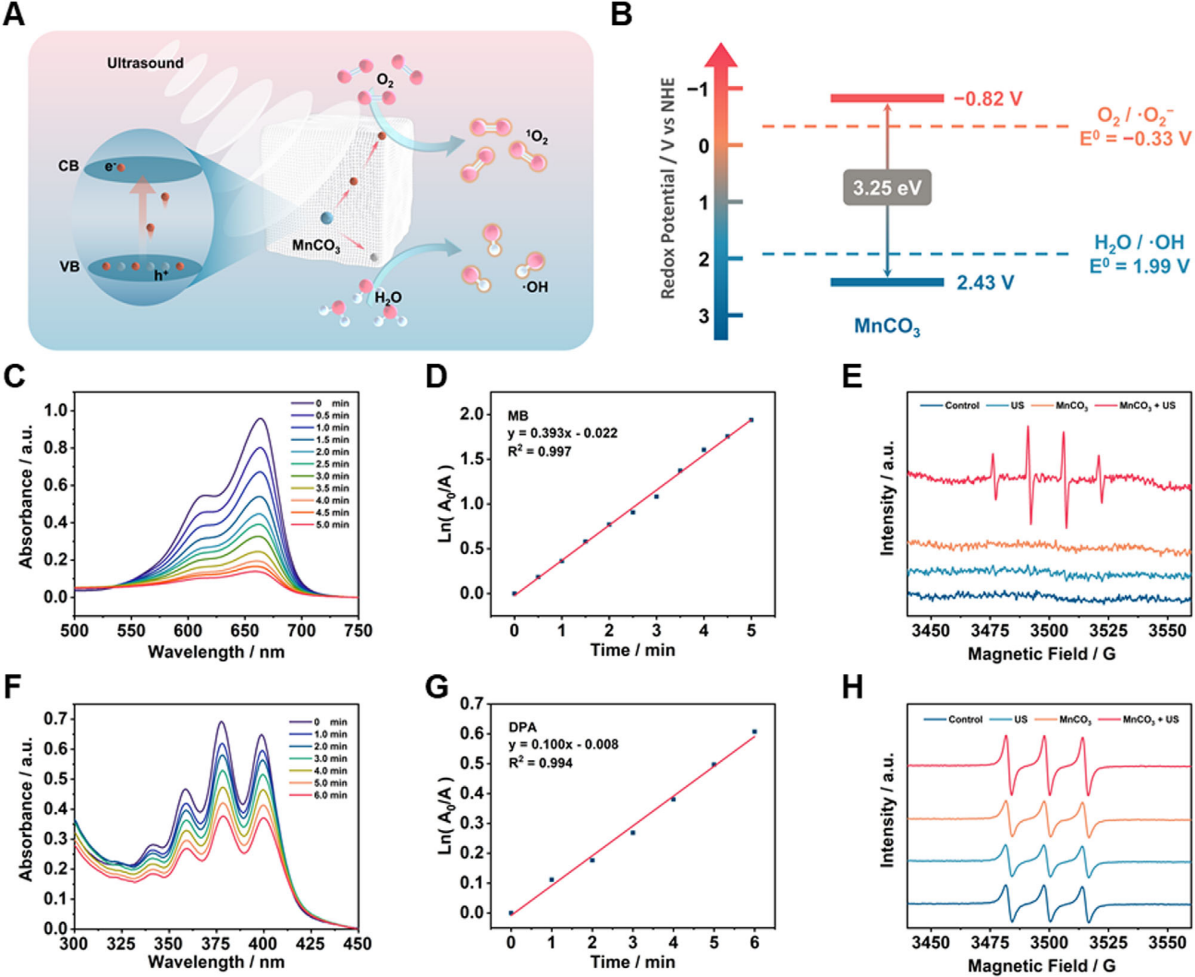
The sonodynamic performance and mechanism of MnCO_3_ NPs. (A) The proposed mechanism of ROS generation by MnCO_3_ NPs under US irradiation. (B) The energy band diagram of MnCO_3_ NPs. Under US (1.0 MHz, 1.5 W·cm^–2^) irradiation, the time‐dependent sono‐degradation of (C) MB and (F) DPA caused by MnCO_3_ NPs. Rate constant for (D) MB and (G) DPA decomposition in the presence of MnCO_3_ NPs. ESR spectra demonstrating (E) •OH and (H) ^1^O_2_ generation of MnCO_3_ NPs with/without US (1.0 MHz, 1.5 W·cm^–2^, 1 min)

Based on the previous reports,^[^ ^27^ ^]^ we speculate MnCO_3_ semiconductor could generate ROS under US stimulation and the mechanism of sonodynamic performance is shown in Figure 2A. US irradiation could trigger the separation of the electron (e^–^)‐hole (h^+^) pairs, then migrate to the surface of MnCO_3_ NPs, and react with surrounding O_2_ and H_2_O to form ^1^O_2_ and •OH, respectively. To verify the above hypothesis, the ultraviolet diffuse reflectance spectrum of solid MnCO_3_ was measured. The band gap was calculated is about 3.25 eV between the valence band (2.43 V) and conduction band (−0.82 V) by plotted Tauc plot of the Kubelka–Munk (KM) function and XPS valence band spectrum (Figure 2B; Figures S7 and S8). The above results proved that MnCO_3_ NPs could achieve electron excitation under US irradiation to further produce ROS. To investigate sonodynamic performance of MnCO_3_ NPs, the chemical probes methylene blue (MB) and 9,10‐diphenanthraquinone (DPA) were carried out to examine the generation of •OH and ^1^O_2_, respectively. Under US irradiation (1.0 MHz, 1.5 W·cm^−2^), the characteristic absorption peak of MB and DPA gradually decrease with time (Figure 2C,F), and the corresponding rate constant was calculated to be 0.393 and 0.100 min^–1^ (Figure 2D,G), respectively. The sono‐degradation data and rate constant of MB and DPA treated with US alone were also provided (Figure S9). In addition, the generation of •OH and ^1^O_2_ was also detected by electron spin resonance (ESR) (Figure 2E,H). By using 5,5‐dimethyl‐1‐pyrroline‐*N*‐oxide (DMPO) and 2,2,6,6‐tetramethylpiperide (TEMP) as capture agents measured for •OH and ^1^O_2_, respectively. At a concentration of 100 µg·mL^−1^ and US radiation (1.0 MHz, 1.5 W·cm^−2^, 1 min), the characteristic peaks of •OH can be clearly observed, and the generation of ^1^O_2_ increased by 162.1% compared with only MnCO_3_ NPs. These results confirmed that MnCO_3_ NPs have excellent ROS production ability as a sonosensitizer.

### 2.3 Microenvironment responsiveness

**FIGURE 3:**
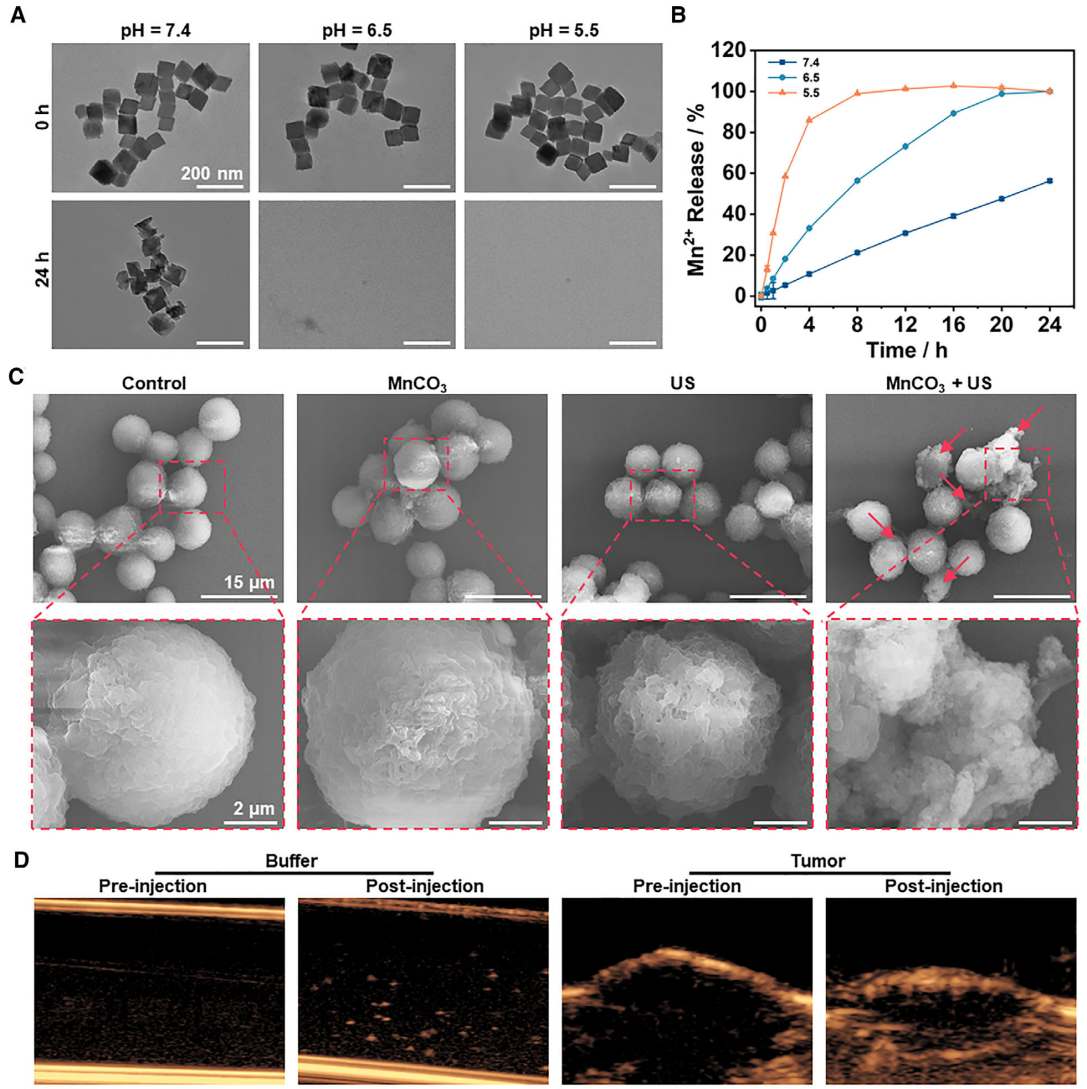
(A) TEM image of MnCO_3_ NPs degradation under different pH. (B) Percentage of manganese ions released from degradation of MnCO_3_ NPs at different pH. (C) SEM images of PBS or MnCO_3_ NPs co‐incubated with 4T1 cells under US (1.0 MHz, 1.5 W·cm^–2^, 2 min, 50% duty cycle) irradiation. (D) Ultrasound imaging of MnCO_3_ NPs in buffer (100 µg·mL^–1^) and tumor (5 mg·kg^–1^)

We systematically researched the acidic tumor microenvironment responsiveness of MnCO_3_ NPs. First, we conducted experiments in different pH buffers. The degradation of MnCO_3_ NPs can be strongly confirmed by TEM morphological observation (Figure 3A) and measurement of Mn^2+^ release (Figure 3B). Under the conditions of pH = 5.5 and 6.5, MnCO_3_ NPs were completely degraded within 8 and 24 h, respectively. After that, to verify the CO_2_ produced by MnCO_3_ NPs in an acidic environment, we used an optical microscope to observe the bubbles in saturated sodium carbonate buffers of different pH. After incubated for 1 h, a large number of bubbles could be observed in pH = 6.5 group (Figure S10). The cell necrosis caused by ultrasonic cavitation of CO_2_ was shown in SEM images (Figure 3C). It can be clearly seen that under the combined action of MnCO_3_ NPs and US, part of the cells was necrotic due to cell membrane damage. Based on the above analysis, we investigated the US imaging contrast function of CO_2_ released from MnCO_3_ NPs. The MnCO_3_ NPs showed obvious imaging signal in the pH = 6.5 buffer and tumor tissue (Figure 3D, Movies S1 and S2). The result proved that MnCO_3_ NPs could act as a good ultrasound contrast agent to guide SDT at specific tumoral acidic pH. We also explored the sonodynamic performance of MnCO_3_ NPs in acidic pH to mimic the ROS production in the tumor microenvironment. As shown in Figure S11, MnCO_3_ can be stimulated by US waves to produce ROS at different pH, but with the decreased of pH, the ROS signal gradually decreased. And the ESR spectrum showed that as the pH decreases, the characteristic peak of paramagnetic manganese gradually increases. These results indicated that the generation of ROS is related to the degradation degree of MnCO_3_ NPs, further indirectly proves the release of Mn^2+^. In addition, MB degradation experiments also confirmed the same conclusion (Figure S12).

### 2.4 SDT and metal ion therapy at the cellular level

**FIGURE 4:**
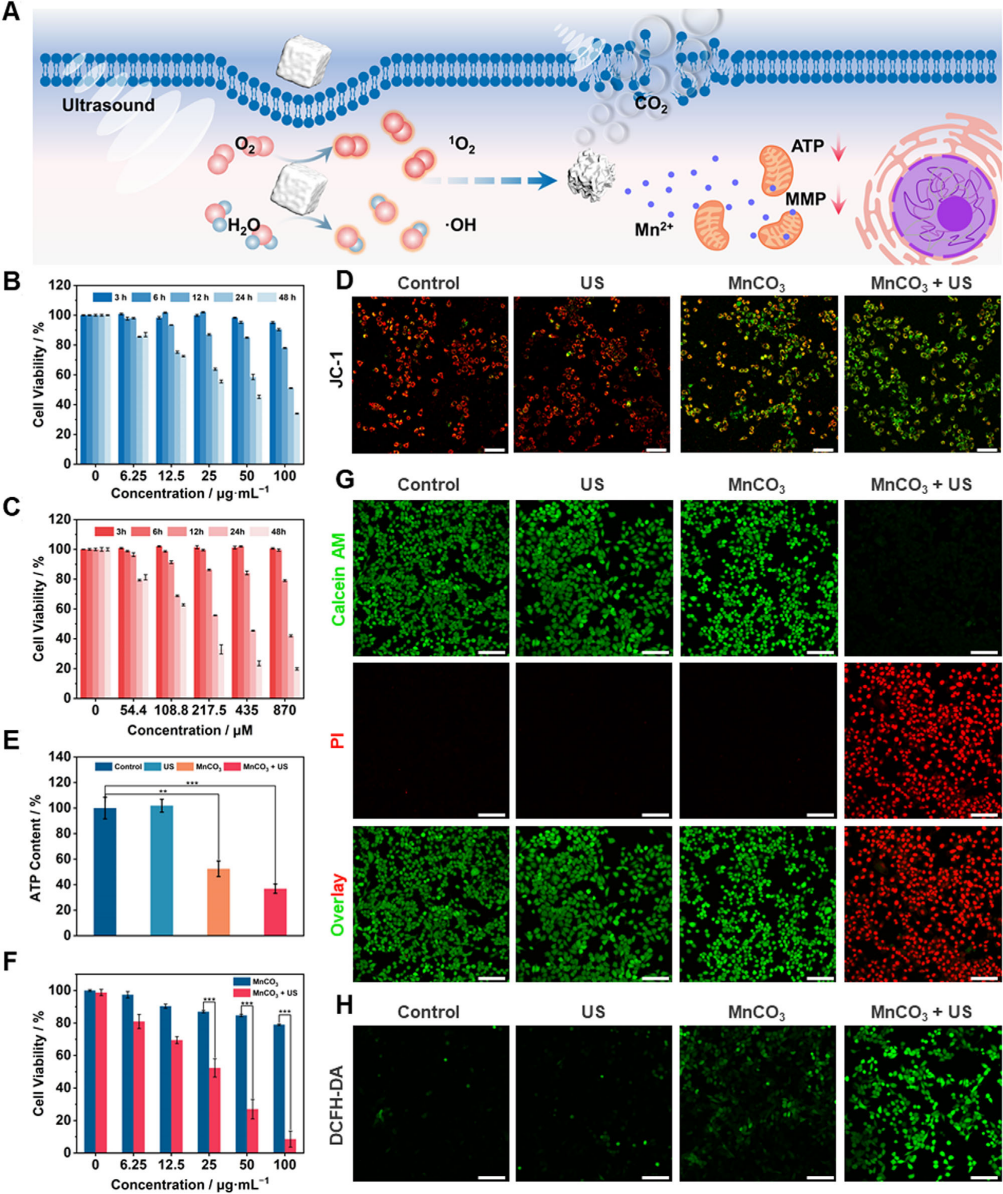
MnCO_3_ NPs mediated SDT and metal ion therapy at the cellular level. (A) Schematic illustration of intracellular treatment of MnCO_3_ NPs. (B) The cell viability of 4T1 cells incubated with different concentrations of MnCO_3_ NPs and different incubation times. (C) The cell viability of 4T1 cells with different concentrations of manganese ion and different incubation times. (D) CLSM images of 4T1 cells mitochondrial membrane potential. MnCO_3_ NPs: 100 µg·mL^–1^, Scale bar = 75 µm. (E) Intracellular ATP content of MnCO_3_ NPs with or without US (1.0 MHz, 1.5 W·cm^–2^, 2 min, 50% duty cycle) irradiation. (F) Cell viability of 4T1 co‐incubation with different concentrations of MnCO_3_ NPs for 3 h and then irradiated by US (1.0 MHz, 1.5 W·cm^–2^, 2 min, 50% duty cycle). (G) CLSM images of 4T1 cells live/dead staining. MnCO_3_ NPs: 100 µg·mL^–1^, Scale bar = 100 µm. (H) CLSM images of 4T1 cells stained with DCFH‐DA for differently treated groups. MnCO_3_ NPs: 100 µg·mL^–1^, Scale bar = 100 µm. Data are presented as mean ±SD (*n* = 5), ns: *p* > 0.05, *p < 0.05, **p < 0.01, ***p < 0.001

Based on the excellent sonodynamic performance and efficient ion release of MnCO_3_ NPs, the therapeutic effect at the cellular level was further evaluated (Figure 4A). The cellular internalization of MnCO_3_ NPs was detected by confocal laser scanning microscopy (CLSM) (Figure S13). After the prepared FITC‐labeled MnCO_3_ NPs and co‐incubation with 4T1 cells for 3 h, obvious endocytosis could be observed. Standard 3‐(4,5‐dimethylthiazol‐2‐yl)‐2,5‐diphenyltetra‐zolium bromide (MTT) assay was utilized to measure the cytotoxicity of MnCO_3_ NPs (Figure 4B). It can be seen that within 12 h, the 4T1 cells co‐incubation with MnCO_3_ NPs at various concentrations showed high viabilities. After 12 h, the cytotoxicity of MnCO_3_ NPs increased significantly, which is presumably due to the cell apoptosis caused by the Mn ions released by the degradation of MnCO_3_ NPs. Therefore, we performed the same experiment with the equivalent Mn ion concentration, and approximate results can be obtained (Figure 4C). And compared with MnCO_3_ NPs, the cell viabilities of 4T1 become lower after co‐incubation with equivalent Mn ion for more than 12 h, which is believed to be caused by the slow release of Mn ions from the MnCO_3_ NPs. To verify the influence of manganese ions on mitochondria, the mitochondrial membrane potential of 4T1 cells after various treatments was evaluated by 5,5′,6,6′‐Tetrachloro‐1,1′,3,3′‐tetraethyl‐imidacarbocyanine (JC‐1) staining (Figure 4D). Under high mitochondrial membrane potential, JC‐1 aggregates are formed and emit red fluorescence; at low mitochondrial membrane potential, it will keep the monomer and emit green fluorescence. By observing the relative levels of red and green fluorescence intensity, it can be seen that the MnCO_3_ group and the MnCO_3_ + US group had strong green fluorescence, indicating that the mitochondria are severely damaged. Based on the above results, the changes of ATP content were detected after different treatments. And it could be seen that compared with the Control group, the ATP content decreased in MnCO_3_ group and the MnCO_3_ + US group (Figure 4E). The above results demonstrated that Mn ion released from MnCO_3_ NPs can affect mitochondria function to induce cell apoptosis. In addition, Figure S14 showed the good biosafety of MnCO_3_ NPs co‐incubated with L929 cells for 12 h. The SDT properties of MnCO_3_ NPs at the cellular level were further assessed; relevant experiments were selected an incubation time within 12 h to exclude the anti‐cancer effect of Mn ions. Under US (1.0 MHz, 1.5 W·cm^−2^, 50% duty cycle, 2 min) irradiation, MnCO_3_ NPs could obviously kill 4T1 cells after 3 h of co‐incubation (Figure 4F), the cell viability of 4T1 cells up to 91.44% at an incubation concentration of 100 µg·mL^−1^. And the result showed under the condition of 6 h co‐incubation, the cell killing effect was slightly weakened due to partial degradation of MnCO_3_ NPs (Figure S15). Moreover, observation of the green/red fluorescence of live/dead staining (calcein AM/PI) by CLSM confirmed the excellent SDT effect of MnCO_3_ NPs (Figure 4G). 2′,7′‐dichlorodihydrofluorescein diacetate (DCFH‐DA) was used to detect the generation of intracellular ROS (Figure 4H). Compared with the Control and the US group, the green fluorescence was slightly increased in the MnCO_3_ group whereas it was significantly observed in the MnCO_3_ + US group. The result indicated that MnCO_3_ NPs could produce efficient ROS to kill cancer cells under US irradiation.

### 2.5 SDT synergistic metal ion therapy in vivo

**FIGURE 5:**
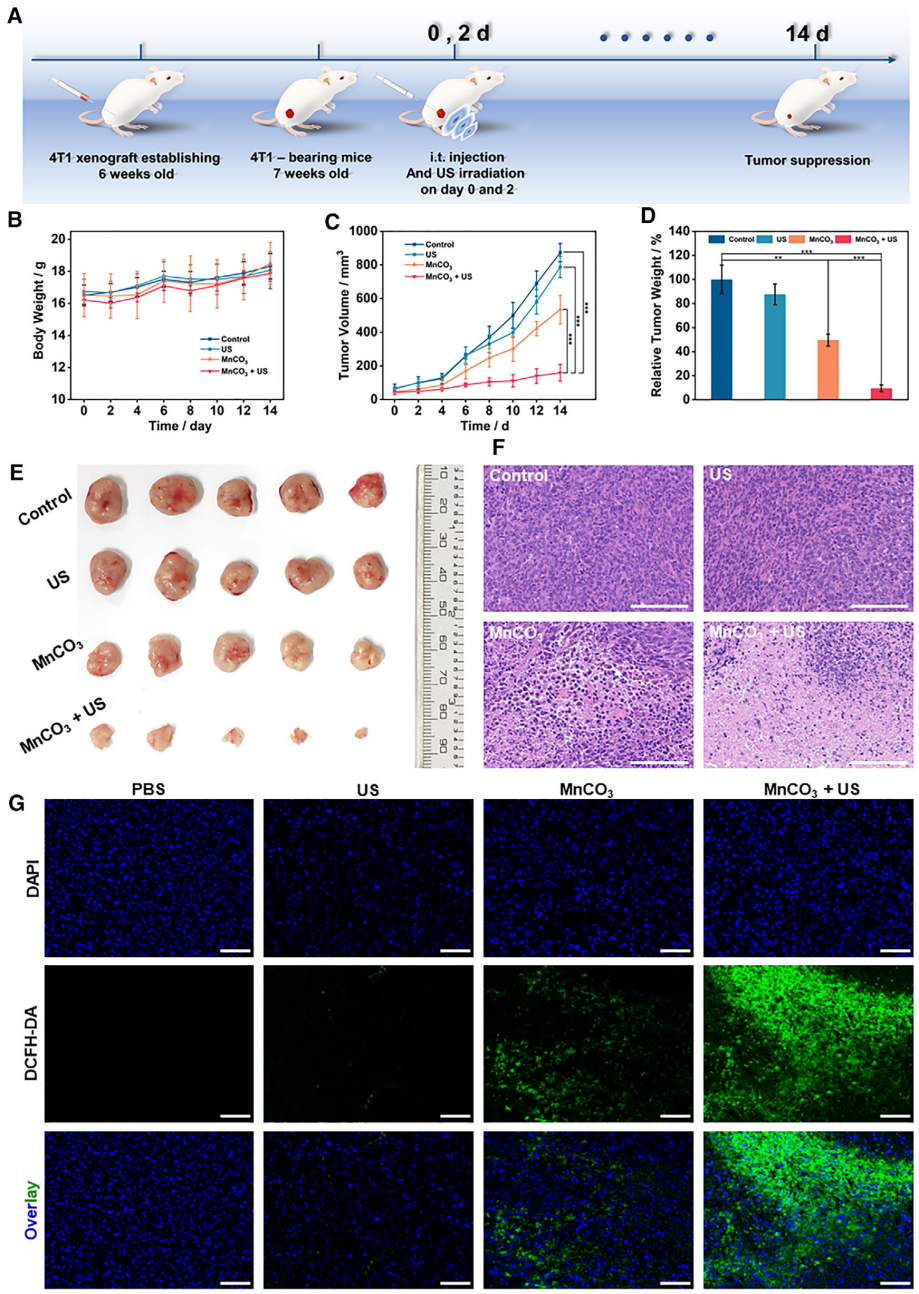
MnCO_3_ NPs mediated SDT synergistic metal ion therapy in vivo. (A) Schematic illustration for tumor treatment. (B) Body weight curve and (C) tumor volume curve of mice with different treatments. (D) Relative average mass of tumors in each group on day 14. (E) Tumor photographs of each group at day 14. (F) H&E‐stained images tumor slices in each group. Scale bar = 100 µm. (G) DAPI‐ and DCFH‐DA‐stained tumor slices in each group. Scale bar = 50 µm. Data are presented as mean ±SD (*n* = 5), ns: *p* > 0.05, *p < 0.05, **p < 0.01, ***p < 0.001

Encouraged by the in vitro results of MnCO_3_ NPs, the in vivo SDT synergistic anti‐cancer was further researched in 4T1 tumor‐xenograft model. The biological safety of MnCO_3_ NPs in vivo was first verified. The hemolysis analysis showed that the hemolysis rate was less than 5% at different concentrations (Figure S16). And an acute toxicity test was carried out, shown in Figure S17. After intravenous injection of 400 mg·kg^–1^ MnCO_3_ NPs, the mice died immediately. After 200 mg·kg^–1^, the mice were in a state of malaise, but did not die. Therefore, the further biosafety assessment was carried out within a concentration of 100 mg·kg^–1^. Through routine blood analysis and body weight recording, no significant difference between control group and different treatments were observed, indicating the good safety profile of MnCO_3_ NPs (Figures S18 and S19). Based on the above analysis, the anti‐tumor properties of MnCO_3_ NPs in vivo were further explored (Figure 5A). The mice were divided into four groups when the tumor volume reached about 50 mm^3^. Intratumoral injected of MnCO_3_ NPs and US irradiation (1.0 MHz, 1.5 W·cm^–2^, 50% duty cycle, 5 min) was performed on day 0 and day 2. During the 14 days of treatment, the tumor volume and the weight of the mice were recorded every two days. The weight of the mice increased slightly during the treatment period, which confirmed that the adverse effect of the dose on the mice was negligible (Figure 5B). At the end of treatment, the MnCO_3_ group had a certain anti‐tumor ability, and the MnCO_3_ +US group had the best therapeutic effect. Their tumor inhibition rates are 50.41% and 90.45%, respectively (Figure 5C–E and Figure S20). Subsequently, hematoxylin and eosin (H&E) staining was performed on the histological sections of the main organs and tumors (Figure 5F and Figure S21). The apoptosis and necrosis of cancer cells could be clearly seen in the MnCO_3_ and MnCO_3_ +US group, and no obvious toxic side effects in the main organs. Furthermore, DCFH‐DA and DAPI staining were utilized to characterize the generation of ROS in vivo. ROS were observed to be produced in both treatment groups, while MnCO_3_ + US group produced more (Figure 5G). These results indicated that MnCO_3_ NPs mediated SDT synergistic metal ion therapy is highly effective anti‐cancer strategy.

## 3 CONCLUSION

In summary, cubic MnCO_3_ NPs were prepared by a simple inverse microemulsion method as a new sonosensitizer for the combined SDT and metal ion therapy. MnCO_3_ NPs have excellent ROS generation ability under US irradiation. Moreover, pH‐responsive MnCO_3_ NPs can degrade in the tumor acidic microenvironment to produce CO_2_ and release Mn^2+^. The generated CO_2_ bubbles caused enhanced cavitation effect under US stimulation, thereby mediating irreversible cell necrosis; the release of Mn^2+^ also induced cell apoptosis through the mitochondrial pathway. In addition, MnCO_3_ NPs could provide US imaging guidance for cancer therapy. According to in vivo experiments, it has been proved that MnCO_3_‐mediated SDT has a high tumor‐inhibiting effect. The multifunctional sonosensitizer is expected to be a promising tool for cancer therapy.

## 4 EXPERIMENTAL SECTION

### 4.1 Materials

Cetyl trimethyl ammonium bromide (CTAB), manganese chloride tetrahydrate, sodium bicarbonate, ammonium bicarbonate, *n*‐pentanol, cyclohexane, sodium acetate, and TEMP were obtained from Dojindo Laboratories. MB and DPA were purchased from Shanghai Macklin Biochemical Co., Ltd (Shanghai, China). Calcein acetoxymethyl ester (Calcein‐AM), methyl thiazolyl tetrazolium (MTT), propidium iodide (PI), DCFH‐DA, BCA protein assay kit, mitochondrial membrane potential detection kit (JC‐1), and H&E were purchased from Beijing Solarbio Science & Technology Co., Ltd. (Beijing, China). ATP assay kit was purchased from Beyotime Biotechnology (China). Dulbecco's modified Eagle medium (DMEM), fetal bovine serum (FBS), phosphate buffered solution (PBS), trypsin‐EDTA solution, penicillin, and streptomycin were purchased from Corning Inc. (New York, USA). All reagents were used as received without any further purification.

### 4.2 Characterization

The morphology and size of the MnCO_3_ NPs were characterized by HT‐7700 transmission electron microscope. DLS measured the size distribution and zeta potential of MnCO_3_ NPs (Zetasizer Nano‐ZS, Malvern Instruments, UK). A 2600 UV–vis–NIR spectrophotometer (SHIMADZU, Japan) was used to obtain the ultraviolet–visible–near‐infrared (UV–vis–NIR) absorption spectrum. The generation of ^1^O_2_ and •OH was determined by ESR spectrometer (Bruker EMXplus). Nicolet 6700 spectrometer obtained the infrared spectra of MnCO_3_ NPs and MnCO_3_. The crystal structure of MnCO_3_ NPs was characterized by powder XRD (XRD‐6000, Japan). XPS was used to evaluate the valence state of Mn. The CO_2_ bubbles were observed by an optical microscope. DJO‐2776 sonicator as an energy converter was applied to generate ultrasound during the treatment. The concentration of Mn^2+^ was determined by inductively coupled plasma‐mass spectrometry (ICP‐MS, Perkin‐Elmer).

### 4.3 Preparation of MnCO_3_ NPs

The preparation method of MnCO_3_ NPs is improved from previous reports. 0.667 g CTAB dispersed in 20 mL cyclohexane and 1 mL *n*‐pentanol, called solution A. 2.668 g CTAB was dispersed in 80 mL cyclohexane and 4 mL *n*‐pentanol, called solution B. Dispersed 3.33 mmoL of manganese chloride tetrahydrate into 0.667 mL of deionized water, which is called solution a. Dispersed 6.33 mmoL sodium bicarbonate 0.33 mmol ammonium bicarbonate into 2.668 mL deionized water, called solution b. Added solution a to solution A to form a transparent emulsion; added the supernatant of solution b to solution B to form a translucent emulsion. Both were stirred for 3 h. Subsequently, the two were mixed and stirred for 1 h to form MnCO_3_ NPs. Alternately washed with ethanol deionized water 5 times and then dried at 30°C for later used.

### 4.4 Preparation of FITC‐labeled MnCO_3_ NPs

15 mg MnCO_3_ NPs and 0.2 mL APTES were dispersed in 15 mL DMF solution, stirred for 24 h, washed with ethanol, and dried to obtain MnCO_3_‐NH_2_. Afterward, 10 mg MnCO_3_‐NH_2_ and 0.5 mL FITC (1 mg·mL^–1^) were dispersed in 9.5 mL ethanol, stirred at room temperature for 24 h, and then centrifuged, washed, and dried before used.

### 4.5 Determination of •OH

10 µL DMPO mixed with 50 µL MnCO_3_ NPs (100 µg·mL^−1^) and then irradiated with US (1.0 MHz, 1.5 W·cm^–2^, 1 min). Determination of the formation of •OH was by ESR spectrometer. In addition, simultaneous detection of the control group, US group, and MnCO_3_ NPs group.

### 4.6 Determination of ^1^O_2_

3 µL TEMP mixed with 100 µL MnCO_3_ NPs (100 µg·mL^−1^) and then irradiated with US (1.0 MHz, 1.5 W·cm^–2^, 1 min). Determination of the formation of ^1^O_2_ was by ESR spectrometer. In addition, simultaneous detection of the control group, US group, and MnCO_3_ NPs group.

### 4.7 Quantitative analysis of •OH

Configured 10 mg·L^–1^ MB, 100 µg·mL^–1^ MnCO_3_ NPs solution, and sonicated 0, 0.5, 1, 1.5, 2, 2.5, 3, 3.5, 4, 4.5, 5 min under ultrasonic conditions (1.5 W, 1 MHz), after which the sample was scanned in the 500−750 nm band with an ultraviolet−visible (UV−vis) spectrophotometer, and the changed in the absorption peak at 655 nm was used to quantify the degradation rate.

### 4.8 Quantitative analysis of ^1^O_2_

Mixed 3.2 mL MnCO_3_ NPs (100 µg·mL^−1^) with 80 µL DPA (1 mg·mL^−1^), and sonicate 0, 1, 2, 3, 4, 5, 6 min under ultrasonic conditions (1.5 W, 1 MHz), after which the sample was scanned in the 300–450 nm band with a UV−vis spectrophotometer, and the changed in the absorption peak at 378 nm was used to quantify the degradation rate.

### 4.9 Degradation of MnCO_3_ NPs **in vitro**

MnCO_3_ NPs degradation experiments were carried out in acetate buffers of different pH (7.4, 6.5, 5.5). Briefly, added 6 mL of MnCO_3_ NPs solution (1 mg·mL^−1^) to the dialysis bag (MWCO = 3.5 k), after sealed, as for 300 mL of different pH buffer systems. Subsequently, 0.5 mL was taken out at the specified time interval, diluted, and used for ICP analysis to test the manganese ion content.

### 4.10 Ultrasonic cavitation observation of cells

Added 100 µL of 25% v/v glutaraldehyde solution to 500 µL of 4T1 cell suspension; immediately US irradiation (1.0 MHz, 1.5 W·cm^–2^, 2 min, 50% duty cycle); washed the cells with PBS; resuspended the cells in 5% (v/v) glutaraldehyde solution. Then washed the cells with 30%, 50%, 70%, 95%, 100% (v/v) ethanol solution, respectively. Finally, observed the cells morphology under SEM.

### 4.11 Cell culture

4T1 cells and L929 cells were cultured in high‐glucose DMEM medium, both containing 10% FBS and 1% penicillin/streptomycin. The cells were cultured in an incubator at 37°C and 5% carbon dioxide.

### 4.12 In vitro cytotoxicity

MTT was used to determine the in vitro cytotoxicity of MnCO_3_ NPs and manganese ions. 4T1 cells were planted in 96‐well plates (10^4^·well^−1^)and incubated for 24 h, then added different concentrations of MnCO_3_ NPs and manganese chloride tetrahydrate (100, 50, 25, 12.5, 6.25, 0 µg·mL^−1^) (870, 435, 217.5, 108.8, 54.5, 0 µM), and MTT assay was used to detect cell viability at a predetermined time. For the researched of the in vitro sonodynamic performance of manganese carbonate, US (1.0 MHz, 1.5 W·cm^−2^, 2 min, 50% duty cycle) was performed during the 3 and 6 h co‐incubation of the materials and the cells, and then MTT was used when the co‐incubation reached 12 h to detect cell viability.

### 4.13 Co‐staining of 4T1 cells with Calcein‐AM and PI

After 4T1 cells were incubated in CLSM‐exclusive culture disk for 24 h, the DMEM medium with MnCO_3_ NPs concentration of 100 µg·mL^−1^ was replaced, and after a total of 3 h incubation, the cells were irradiated with US (1.0 MHz, 1.5 W·cm^−2^, 2 min, 50% duty cycle). After 9 h, the cells were stained with PI and Calcein‐AM. Then, observed by the CLSM. The Control group, the US group, and the MnCO_3_ NPs group performed the same operation.

### 4.14 ROS assay at a cellular level

Using DCFH‐DA to detect intracellular ROS. After 4T1 cells were incubated in CLSM‐exclusive culture disk for 24 h, the DMEM medium with MnCO_3_ NPs concentration of 100 µg·mL^−1^ was replaced, and after a total of 3 h, the cells were irradiated with US (1.0 MHz, 1.5 W·cm^−2^, 2 min, 50% duty cycle). Finally, detected with DCFH‐DA molecular probe and observe by CLSM.

### 4.15 Observation of mitochondrial membrane potential and detection of ATP content

The JC‐1 mitochondrial membrane potential detection kit was used for mitochondrial membrane potential observation experiments, and the ATP detection kit and BCA method trace protein detection kit were used for ATP content detection. After the cells were incubated for 24 h, the DMEM medium with MnCO_3_ NPs concentration of 100 µg·mL^−1^ was replaced, and after a total of 3 h of incubation, the cells were irradiated with US (1.0 MHz, 1.5 W·cm^−2^, 2 min, 50% duty cycle). JC‐1 was used after 9 h and observed by CLSM. After processing with the ATP detection kit and the BCA trace protein detection kit, the absorbance change was detected with a microplate reader.

### 4.16 Hemolysis assay

The red blood cells were collected from BALB/c mice to evaluate the hematotoxicity of MnCO_3_ NPs in vitro. First, the red blood cells were collected by centrifugation at 4°C, washed three times with PBS. Subsequently, the MnCO_3_ NPs were dispersed in PBS with a series concentration (3.125, 6.25, 12.5, 25, 50, 100, and 200 µg·mL^−1^), followed by adding into the red blood cells, respectively. Simultaneously, the positive and negative groups were tested with deionized water and PBS, respectively. The mixture was kept standing at room temperature for 1 h and collected by centrifugation at 3000 rpm for 10 min. The supernatant was collected and measured the absorbance at 570 nm. The hemolysis ratio was calculated by the following formula: hemolysis rate (%) = (sample absorption − negative control absorption) / (positive control absorption − negative control absorption) × 100%.

### 4.17 Tumor model

Female SPF BALB/c mice (6 weeks) were purchased from Beijing Vital River Laboratory Animal Technology Co., Ltd. All the xenograft 4T1 tumor models were established by injecting 0.05 mL of 4T1 cells (1 × 10^4^, dispersed in PBS) into the SPF BALB/c female mice subcutaneous.

### 4.18 In vivo biological safety of MnCO_3_ NPs

BALB/c mice were injected with MnCO_3_ NPs (25, 50, 100 mg·kg^−1^) through the tail vein. Body weight is measured every two days to evaluate the biological safety in vivo. Afterward, the mice were euthanized at a set time; blood was collected for a complete serum biochemical test.

### 4.19 MnCO_3_ NPs treatment in vivo

The 4T1 tumor‐bearing mice were randomly divided into four groups (*n* = 5): (1) control group, only injected with PBS; (2) US group (injected with PBS, 1.0 MHz, 1.5 W·cm^−2^, 5 min, 50% duty cycle); (3) MnCO_3_ NPs group (5 mg·kg^−1^); (4) MnCO_3_ NPs + US group (5 mg·kg^−1^, 1.0 MHz, 1.5 W·cm^−2^, 5 min, 50% duty cycle). Each group was injected intratumorally on the 0th and second day, and the US group and the MnCO_3_ + US group were treated with US 10 min after the injection. The body weight and tumor volume of the mice were recorded every two days. The tumor volume formula is as follows: volume = (tumor length) × (tumor width)^2^/2. After the 14 days course of treatment, all mice were euthanized, and the tumors and major organs were collected for H&E staining and analyzed.

## CONFLICT OF INTEREST

Huiyu Liu is a member of the *Exploration* editorial board. The authors declare no competing interests.

## Supporting information



SUPPORTING INFORMATION

SUPPLEMENTAL VIDEO 1

SUPPLEMENTAL VIDEO 2
