# Supplementary material for: Manganese carbonate nanoparticles‐mediated mitochondrial dysfunction for enhanced sonodynamic therapy
Source: Exploration (Beijing). 2021 Sep 30;1(2):20210010. doi: 10.1002/EXP.20210010 (PMC10190974; doi:10.1002/EXP.20210010)
Supplement: Supplementary file 1 — SUPPORTING INFORMATION [file EXP2-1-20210010-s002.docx]

Supporting information

**Manganese carbonate nanoparticles-mediated mitochondrial dysfunction for enhanced sonodynamic therapy**

Haoyuan Zhang^#^, Xueting Pan ^#^, Qingyuan Wu, Juan Guo, Chaohui Wang, Huiyu Liu*

H. Y. Zhang, X. T. Pan, Q. Y. Wu, J. Guo, C. H. Wang, Prof. H. Y. Liu

Beijing Advanced Innovation Center for Soft Matter Science and Engineering, State Key Laboratory of Organic-Inorganic Composites, Beijing Laboratory of Biomedical Materials, Bionanomaterials & Translational Engineering Laboratory, Beijing Key Laboratory of Bioprocess, Beijing University of Chemical Technology, Beijing 100029, P.R. China

E-mail: liuhy@mail.buct.edu.cn


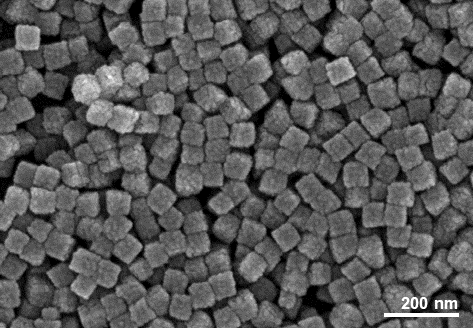


**Figure S1.** The SEM image of MnCO_3_ NPs.

**
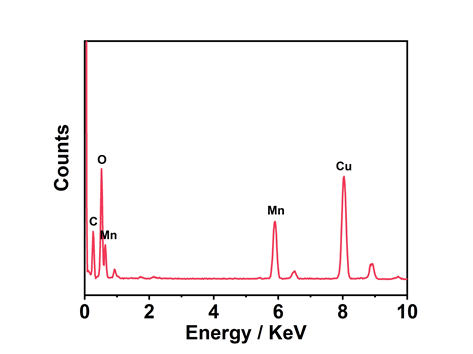
**

**Figure S2.** EDS spectrum of MnCO_3_ NPs.


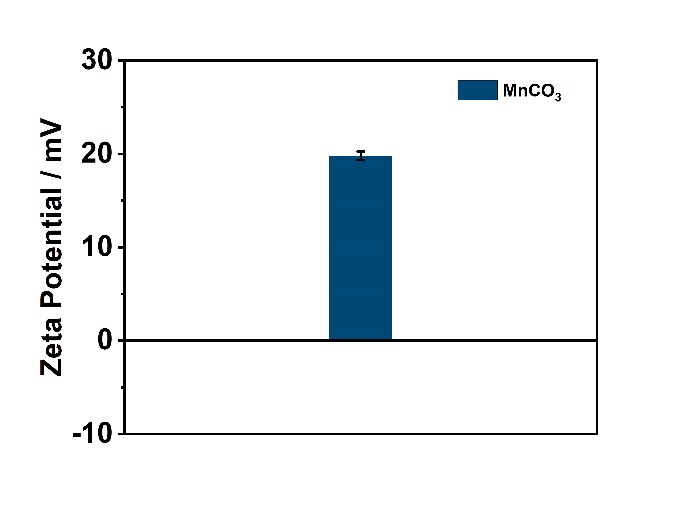


**Figure S3.** The zeta potential of MnCO_3_ NPs.


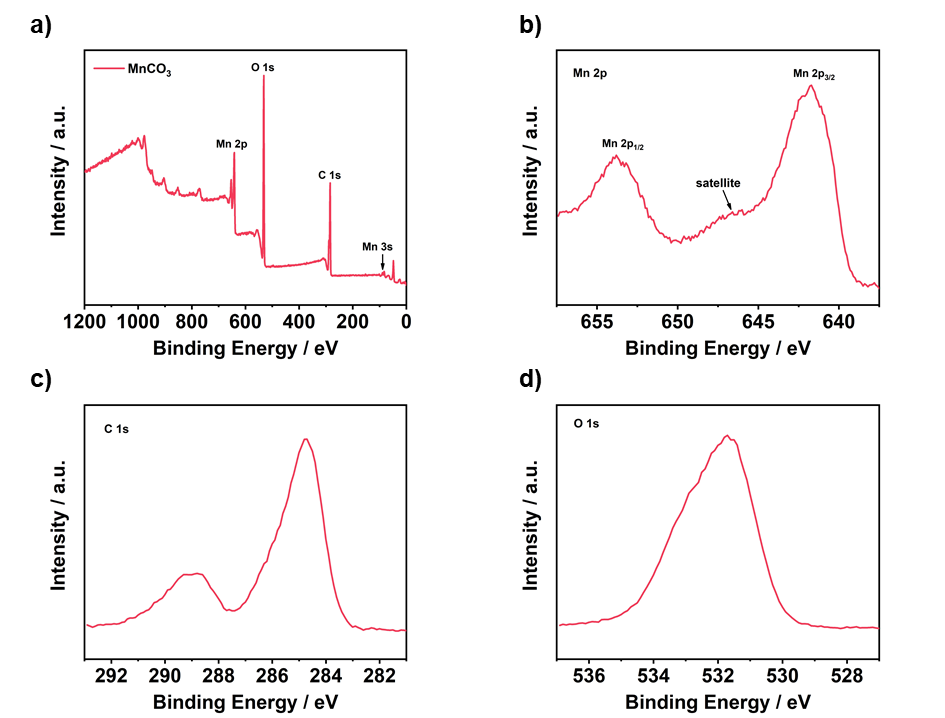


**Figure S4.** XPS of a) survey spectrum, b) Mn 2p, c) C 1s and d) O 1s in MnCO_3_ NPs.

**
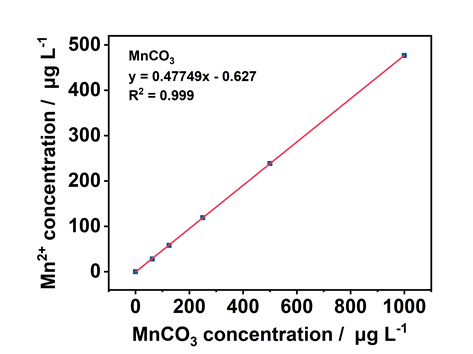
**

**Figure S5.** Manganese ion content in MnCO_3_ NPs measured by ICP-MS.


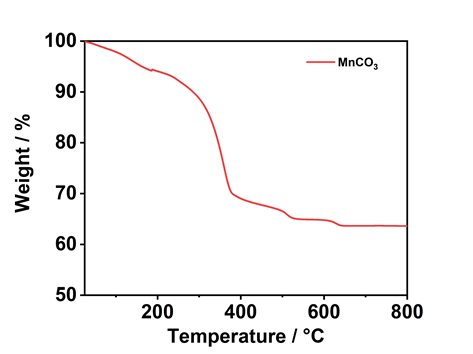


**Figure S6.** TG curves for MnCO_3_ NPs scanned at heating rate of 10 °C min^−1^ in air atmosphere.


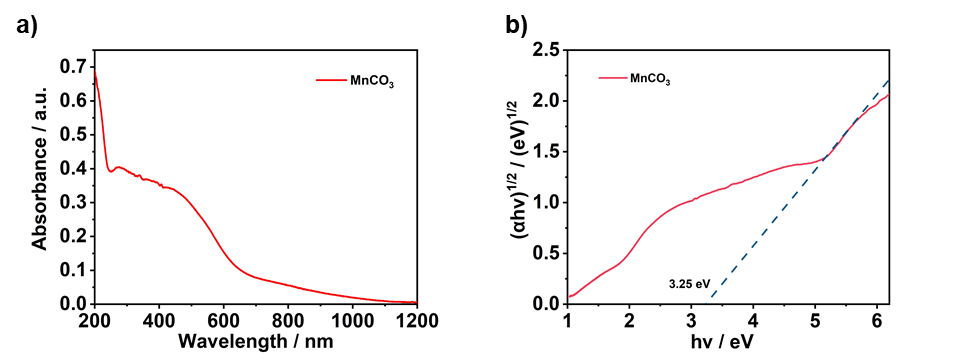


**Figure S7.** a) UV–vis–NIR diffuse reflectance spectrum and b) optical bandgap of MnCO_3_ NPs.


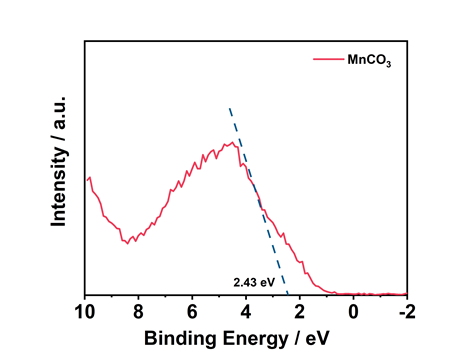


**Figure S8.** XPS VB spectra of MnCO_3_ NPs.


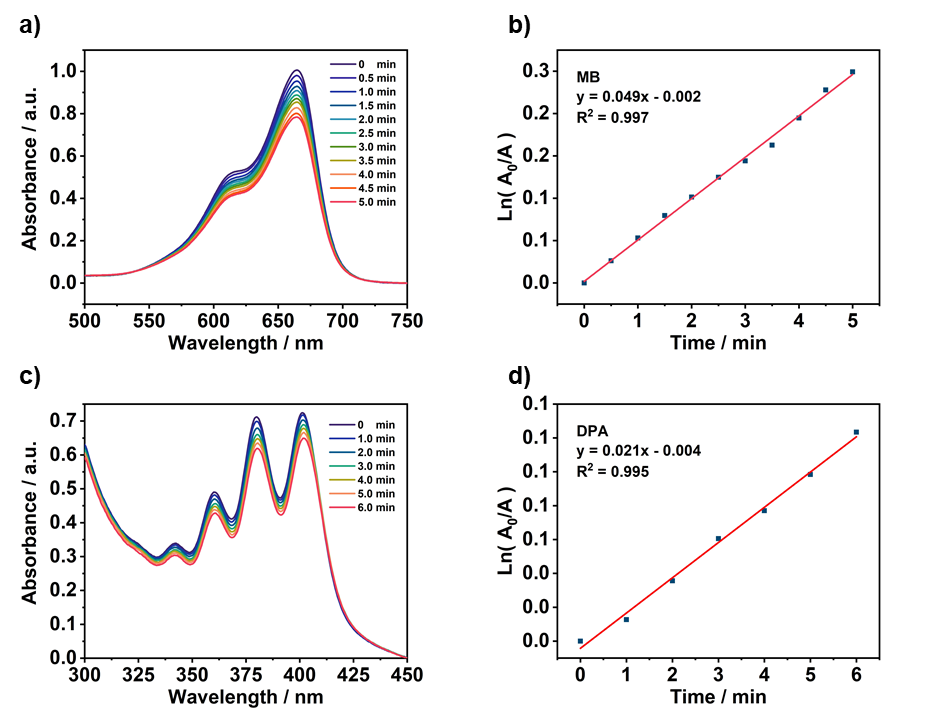


**Figure S9.** Time-dependent sono-degradation of a) MB and c) DPA under US irradiation (1.0 MHz, 1.5 W cm^-^2). Rate constant for b) MB and d) DPA decomposition.


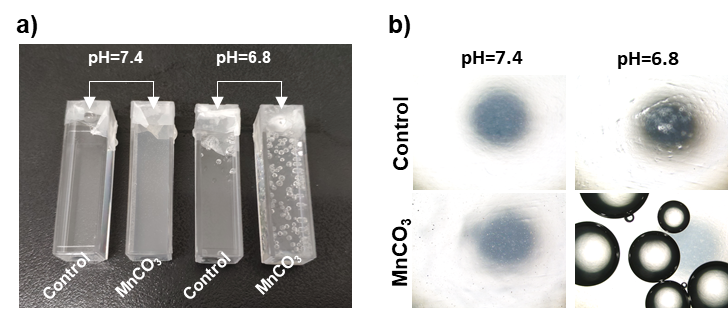


**Figure S10.** a) Photographs and b) optical microscope images of CO_2_ produced by MnCO_3_ NPs in saturated carbonic acid solutions of different pH.


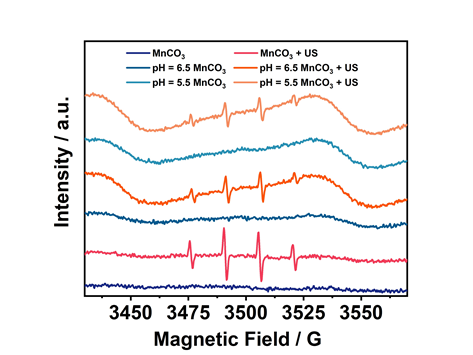


**Figure S11.** ESR spectra demonstrating ·OH generation of MnCO_3_ NPs with or without US (1.0 MHz, 1.5 Wcm^−2^, 1min) in different pH buffers.


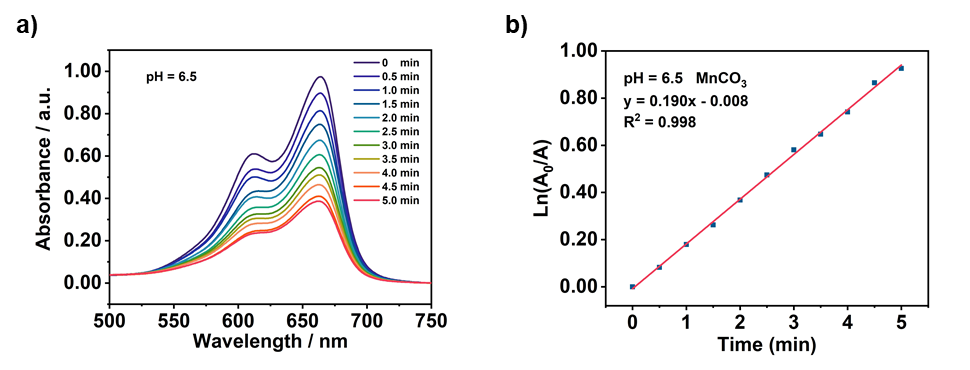


**Figure S12.** a) Time-dependent sono-degradation of MB caused by MnCO_3_ NPs under US (1.0 MHz, 1.5 Wcm^-2^) irradiation in pH 6.5 buffer. b) Rate constant for MB decomposition in the presence of MnCO_3_ NPs.


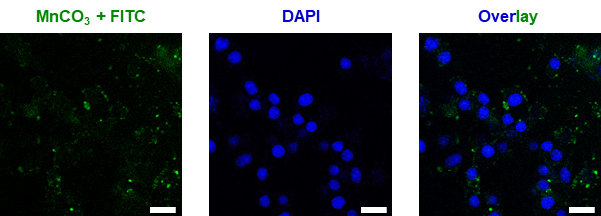


**Figure S13.** CLSM images of 4T1 cells incubated with FITC-MnCO_3_ NPs for 3 h. Scale bar = 25 μm.


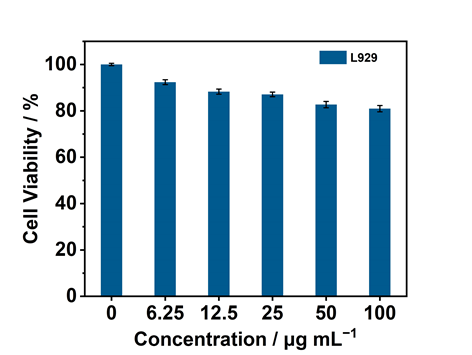


**Figure S14.** Cell viability of L929 cells treated with MnCO_3_ NPs for 12h.


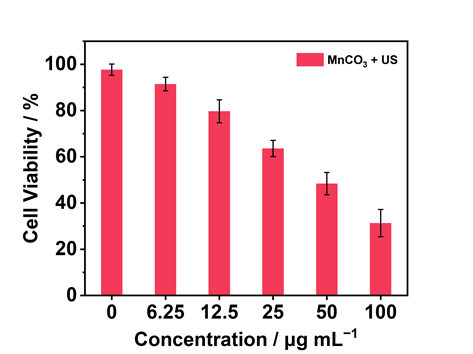


**Figure S15.** Cell viability of 4T1 co-incubation with different concentrations of MnCO_3_ NPs for 6 h and then irradiated by US (1.0 MHz, 1.5 W cm^−2^, 1 min, 50% duty cycle).


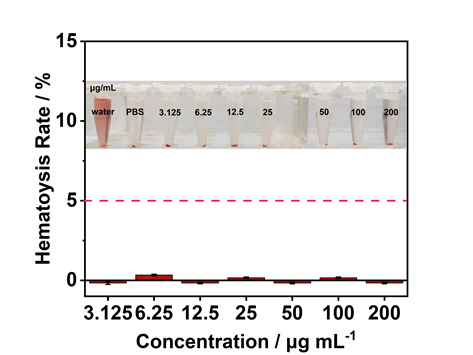


**Figure S16.** Hematolysis rate of MnCO_3_ NPs at different concentrations.


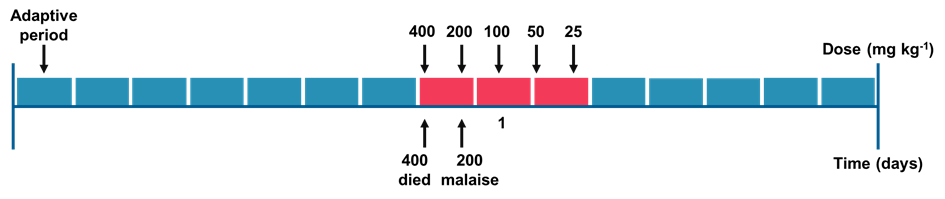


**Figure S17.** The observation and process of systemic toxicity in mice with different doses (25, 50, 100, 200 and 400 mg kg^-1^) of MnCO_3_ NPs intravenously.


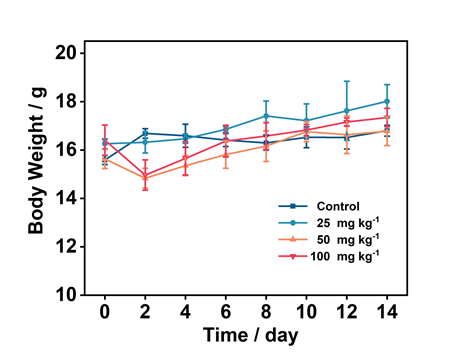


**Figure S18.** The body weight curves of mice after intravenous injection of MnCO_3_ NPs with different doses (25, 50 and 100 mg kg^-1^).


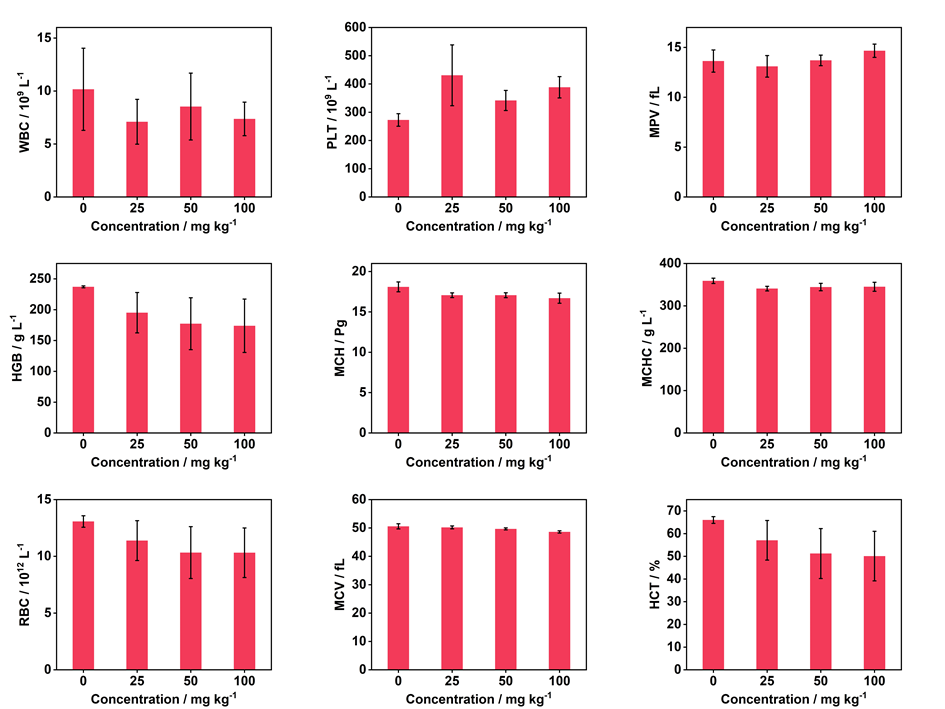


**Figure S19.** Blood biochemical and hematological analysis of different doses (25, 50 and 100 mg kg^-1^) of MnCO_3_ NPs on the 14th day after intravenous injection.


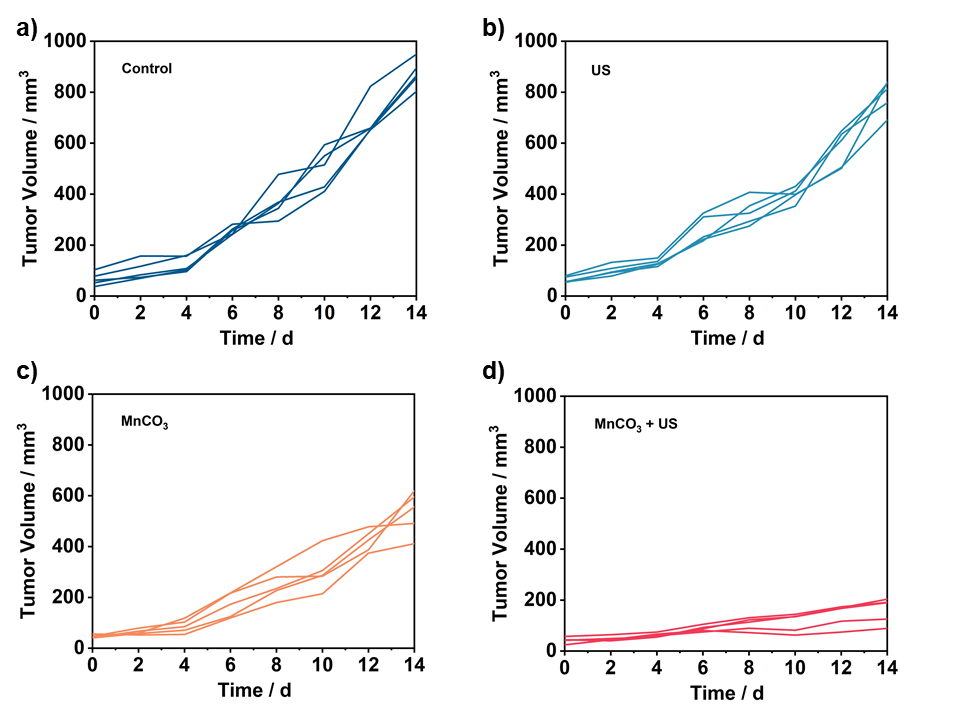


**Figure S20.** Tumor volume curves of single mice with different treatments.


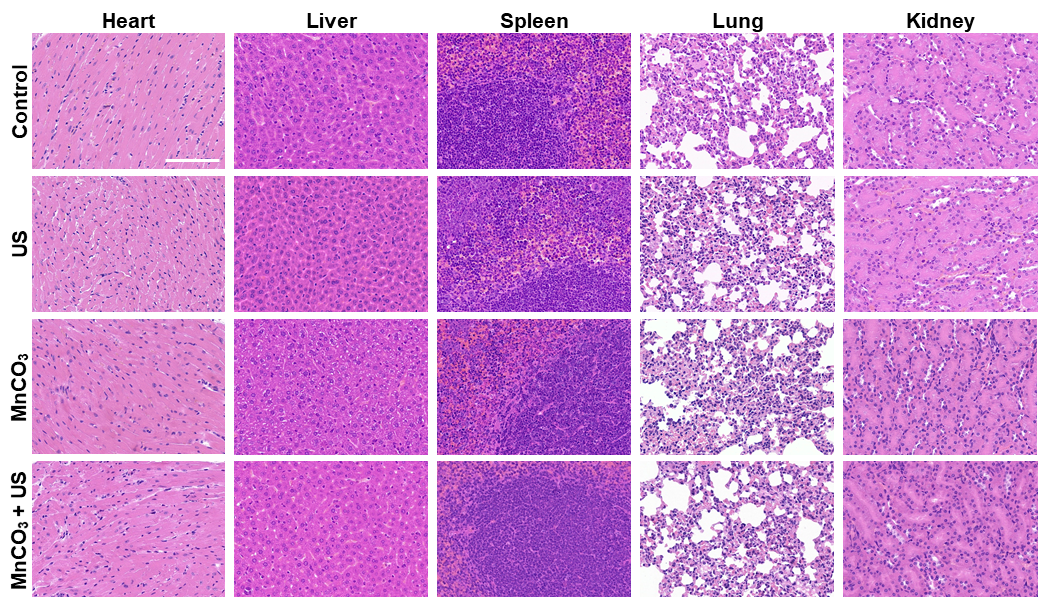


**Figure S21.** H&E stained images of main organs after different treatments. Scale bar = 100 µm.
